# Randomized Comparison of Cardiotoxicity With 60 Versus 90 mg Daunorubicin in AML Induction Therapy

**DOI:** 10.1002/ajh.70160

**Published:** 2026-01-05

**Authors:** Stefan Markus Dendorfer, Katharina Schmidt‐Brücken, Michael Kramer, Björn Steffen, Christoph Schliemann, Jan‐Henrik Mikesch, Nael Alakel, Regina Herbst, Mathias Hänel, Maher Hanoun, Martin Kaufmann, Barbora Weinbergerova, Kerstin Schäfer‐Eckart, Tim Sauer, Andreas Neubauer, Andreas Burchert, Claudia D. Baldus, Jolana Mertová, Edgar Jost, Dirk Niemann, Jan Novák, Stefan W. Krause, Sebastian Scholl, Andreas Hochhaus, Gerhard Held, Tomáš Szotkowski, Andreas Rank, Christoph Schmid, Lars Fransecky, Sabine Kayser, Markus Schaich, Frank Fiebig, Annett Haake, Johannes Schetelig, Jan Moritz Middeke, Friedrich Stölzel, Uwe Platzbecker, Christian Thiede, Carsten Müller‐Tidow, Wolfgang E. Berdel, Gerhard Ehninger, Jiri Mayer, Hubert Serve, Martin Bornhäuser, Christoph Röllig

**Affiliations:** ^1^ Department of Internal Medicine I University Hospital TU Dresden Dresden Germany; ^2^ Cognium GmbH Bern Switzerland; ^3^ Department of Internal Medicine II University Hospital Frankfurt Frankfurt Germany; ^4^ Department of Medicine A University Hospital Münster Münster Germany; ^5^ Department of Internal Medicine III Chemnitz Hospital Chemnitz Germany; ^6^ Department of Hematology University Hospital Essen, University of Duisburg‐Essen Essen Germany; ^7^ Department of Hematology Robert‐Bosch‐Krankenhaus Stuttgart Germany; ^8^ Department of Internal Medicine, Hematology and Oncology University Hospital Brno Brno Czech Republic; ^9^ Department of Internal Medicine V Nuremberg Hospital North, Paracelsus Medical University Nuremberg Germany; ^10^ Department of Internal Medicine V University of Heidelberg Heidelberg Germany; ^11^ Department of Internal Medicine, Hematology, Oncology and Immunology University Hospital Marburg Marburg Germany; ^12^ Department of Hematology and Oncology University Hospital Schleswig‐Holstein, Campus Kiel Kiel Germany; ^13^ Institute of Hematology and Blood Transfusion Prague Czech Republic; ^14^ Department of Hematology, Oncology, Hemostaseology and Stem Cell Transplantation University Hospital Aachen & CIO ABCD Aachen, RWTH Aachen Aachen Germany; ^15^ Department of Hematology, Oncology and Palliative Care Gemeinschaftsklinikum Mittelrhein Koblenz Germany; ^16^ Department of Haematology, 3rd Faculty of Medicine Charles University and University Hospital Kralovske Vinohrady Prague Czech Republic; ^17^ Department of Internal Medicine V University Hospital Erlangen Erlangen Germany; ^18^ Department of Internal Medicine University Hospital Jena Jena Germany; ^19^ Department of Internal Medicine I Westpfalzklinikum Kaiserslautern Germany; ^20^ Department of Hemato‐Oncology Palacký University Olomouc Czech Republic; ^21^ II. Medical Clinic University Hospital Augsburg Augsburg Germany; ^22^ Department of Internal Medicine I, Hematology and Cellular Therapy University Hospital Leipzig Leipzig Germany; ^23^ NCT Trial Center, National Center for Tumor Diseases, German Cancer Research Center (DKFZ) Heidelberg Germany; ^24^ Institute of Transfusion Medicine and Immunology, Medical Faculty Mannheim Heidelberg University Mannheim Germany; ^25^ Department of Hematology, Oncology and Palliative Care Rems‐Murr‐Klinikum Winnenden Germany; ^26^ Agendix GmbH Dresden Germany

**Keywords:** acute myeloid leukemia, anthracycline chemotherapy, cardiotoxicity, daunorubicin dosing, high‐sensitivity troponin T

## Abstract

Anthracyclines are an essential component of induction therapy for acute myeloid leukemia (AML), but their optimal dosing and the associated risk for cardiotoxicity remain under debate. The DaunoDouble trial compared daunorubicin at 60 (Dauno60) versus 90 mg/m^2^ (Dauno90) in combination with cytarabine (100 mg/m^2^ for 7 days) in newly diagnosed AML patients aged 18–65 years. Cardiac function was assessed by left ventricular ejection fraction (LVEF) and cardiac biomarkers (high‐sensitivity troponin T [hsTnT], NT‐proBNP) before treatment and on Day 15 in 317 randomized patients. Median LVEF declined significantly from 65% [IQR 60%–68.5%] to 61% [IQR 58%–67.8%] across all patients (*p* < 0.01), without significant differences between treatment arms. NT‐proBNP levels measured after induction therapy correlated negatively with LVEF at the same time point (*ρ* = −0.24, *p* = 0.02), but did not change significantly during induction—neither between Day 1 and 15 nor between treatment arms. HsTnT levels increased significantly from 5 [IQR 4–8] to 8 ng/L [IQR 6–14] across all patients (*p* < 0.01), with higher post‐induction values in the Dauno90 group (9.5 ng/L [IQR 7–14]) compared to Dauno60 (7 ng/L [IQR 5–14]; *p* < 0.01). In exploratory subgroup analyses, post‐induction hsTnT levels were also significantly higher in patients with obesity and arterial hypertension. These findings provide evidence of a dose‐dependent cardiotoxic effect of daunorubicin, already apparent at standard induction doses, and underscore the importance of early cardiac monitoring. Long‐term follow‐up will be essential to determine the clinical significance of these early changes.

**Trial Registration:** ClinicalTrials.gov identifier: NCT02140242

## Graphical abstract

Early cardiac biomarker monitoring in AML induction therapy showed significantly higher hsTnT levels after daunorubicin 90 mg/m^2^ compared with 60 mg/m^2^, indicating a dose‐dependent early myocardial effect.

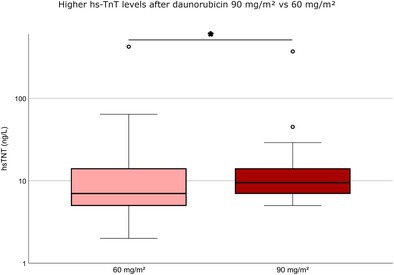

## 1 Introduction

Standard induction therapy for acute myeloid leukemia (AML) consists of a 7‐day continuous infusion of cytarabine combined with a 3‐day administration of an anthracycline. However, the optimal anthracycline dose remains uncertain. The prospective, randomized, multicenter DaunoDouble trial addressed this question by comparing daunorubicin at 60 (Dauno60) versus 90 mg/m^2^ (Dauno90) within the standard 7 + 3 regimen in newly diagnosed AML patients and found no significant differences in efficacy or general tolerability of the two doses [1]. Anthracycline‐induced cardiotoxicity is a well‐recognized complication, particularly with cumulative or high‐dose exposure, but the extent of early, dose‐dependent effects during induction remains unclear [2]. Therefore, we implemented specific cardiac monitoring during the first induction cycle of the trial. This analysis reports the results of left ventricular ejection fraction (LVEF), high‐sensitivity troponin T (hsTnT), and NT‐proBNP as markers of dose‐dependent early cardiac effects.

## 2 Methods

### 2.1 Patients

The DaunoDouble trial (ClinicalTrials.gov identifier NCT02140242) enrolled patients aged 18–65 years with newly diagnosed AML, classified according to the WHO 2008/2016 criteria, excluding cases of acute promyelocytic leukemia. Additional inclusion criteria comprised an Eastern Cooperative Oncology Group (ECOG) performance status of 0–2, adequate hepatic and renal function, no active infection, and a LVEF of ≥ 50%. The DaunoDouble trial was conducted in accordance with the Declaration of Helsinki and approved by the ethics committee of the Technische Universität Dresden, Germany. All participating study centers obtained local ethical approval before patient enrollment, and all patients provided written informed consent prior to inclusion. Participants received induction therapy consisting of 7 days of continuous cytarabine (100 mg/m^2^) and 3 days of daunorubicin, administered at randomized doses of either 60 or 90 mg/m^2^. Following a planned interim analysis, the steering committee discontinued randomization due to a clinically irrelevant difference in good responders. Repeated cardiac monitoring was likewise no longer mandated as part of the study protocol. This subanalysis evaluates the final dataset of the initial study cohort, comprising patients who had been randomized to receive either 60 or 90 mg/m^2^ of daunorubicin and underwent predefined longitudinal cardiac monitoring.

### 2.2 Measurements

LVEF, hsTnT and NT‐proBNP were measured within 3 days before Day 1 of standard 7 + 3 induction therapy and on Day 15. LVEF was assessed using transthoracic two‐dimensional echocardiography at the local treatment centers. hsTnT and NT‐proBNP concentrations were measured using the Elecsys Troponin T high‐sensitive and Elecsys NT‐proBNP assays (Roche Diagnostics, Mannheim, Germany) in locally accredited laboratories. According to the manufacturer's specifications, the 99th percentile upper reference limit for hsTnT is 14 ng/L, while NT‐proBNP values below 125 ng/L are generally considered normal in individuals under 75 years of age.

### 2.3 Statistical Analysis

Unless otherwise specified, all analyses were performed using quantitative continuous data. The main outcome measure of this substudy was LVEF on Day 15 of the first induction cycle. The Wilcoxon test was used for pairwise comparisons over time, while the Mann–Whitney test assessed treatment arm differences at Day 15. Additionally, the temporal progression and group differences of troponin T and NT‐proBNP at Day 15 were analyzed using the same test methods. Genetic risk stratification followed the European LeukemiaNet 2017 (ELN) classification; post‐induction cardiac parameters were compared between the resulting risk groups using the nonparametric Kruskal–Wallis test. Relapse‐free survival was analyzed using univariable Cox proportional hazards regression. Spearman rank correlation was used to explore associations between continuous variables; 95% confidence intervals (95% CI) were reported for correlation coefficients. Within‐subject analyses (e.g., pre‐ to post‐intervention) were restricted to patients with complete paired data (listwise deletion). For between‐group comparisons at the post‐intervention time point, all patients with available measurements were included (available‐case approach). The overall significance level was set at 0.05 (two‐sided), and analyses were performed using IBM SPSS Statistics for Windows, Version 24.0 (IBM Corp., Armonk, NY, USA).

## 3 Results

A total number of 317 patients were randomized to receive either 60 (*n* = 160) or 90 mg/m^2^ (*n* = 157) of daunorubicin. Baseline clinical and leukemia characteristics were comparable between the treatment arms (Tables 1 and 2). Of the 317 randomized patients, 309 received induction therapy (Dauno60, *n* = 155; Dauno90, *n* = 154). Among the remaining eight patients, reasons for not initiating study treatment were revised diagnosis (*n* = 2), serious pretreatment medical complications (*n* = 2), protocol deviation (*n* = 1), and unknown reasons (*n* = 3).

**TABLE 1:** Clinical baseline characteristics of patients from the initial DaunoDouble cohort who underwent early cardiac monitoring, stratified by daunorubicin dose.

Characteristic	Total (*n* = 317)	Dauno60 (*n* = 160)	Dauno90 (*n* = 157)
Age, years, median (range)	51 (18–60)	50 (18–60)	52 (19–60)
Female sex, *n* (%)	159 (50.3)	86 (53.5)	74 (47.1)
ECOG performance status, *n* (%)			
0–1	303 (95.6)	149 (94.9)	151 (96.2)
≥ 2	14 (4.4)	8 (5.1)	6 (3.8)
BMI (kg/m^2^), median (range)	25.8 (13.6–54.6)	25.4 (16.4–52.2)	25.8 (13.6–54.6)
BMI ≥ 30, *n* (%)	60 (19)	35 (22)	25 (16)
Hypertension, *n* (%)	72 (23)	32 (20)	40 (25)
Diabetes mellitus, *n* (%)	18 (6)	10 (6)	8 (5)
LVEF (%), median (range)	65 (48–86)	65 (48–84)	65 (50–86)
hsTnT (ng/L), median (range)	6 (2–88)	6 (2–52)	6 (2–88)
NT‐proBNP (ng/L), median (range)	138.9 (6.0–4097.0)	127.1 (6.0–4097.0)	142.3 (8.7–2975.0)

*Note*: Data are presented using an available‐case approach, including all non‐missing observations.

Abbreviations: BMI, body mass index; ECOG, Eastern Cooperative Oncology Group; hsTnT, high‐sensitivity troponin T; LVEF, left ventricular ejection fraction; NT‐proBNP, N‐terminal pro‐B‐type natriuretic peptide.

**TABLE 2:** Leukemia‐related baseline characteristics of patients from the DaunoDouble cohort who underwent early cardiac monitoring, stratified by daunorubicin dose.

	Total (*n* = 317)	Dauno60 (*n* = 160)	Dauno90 (*n* = 157)
ELN 2017 risk group, *n* (%)			
Favorable	110 (36.7)	52 (34.7)	58 (38.7)
Intermediate	140 (46.7)	72 (48.0)	68 (45.3)
Adverse	50 (16.7)	26 (17.3)	24 (16.0)
Missing	17	10	7
NPM1, *n* (%)			
Mutated	117 (40.5)	57 (39.3)	60 (41.7)
Wild‐type	172 (59.5)	88 (60.7)	84 (58.3)
Missing	28	15	13
FLT3‐ITD, *n* (%)			
Present	54 (20.1)	23 (17.0)	31 (23.1)
Absent	215 (79.9)	112 (83.0)	103 (76.9)
Missing	48	25	23

*Note*: All between‐group comparisons (Dauno60 vs. Dauno90) were nonsignificant by *χ* ^2^ test.

Abbreviations: FLT3, FMS‐like tyrosine kinase 3; ITD, internal tandem duplication; NPM1, nucleophosmin 1.

Eight patients died within the first 30 days of treatment (Dauno60, *n* = 3; Dauno90, *n* = 5), corresponding to early mortality rates of 2% and 3%, respectively. None of these deaths were attributed to a cardiac cause; six were due to infection‐related complications. In total, 21 patients (Dauno60, *n* = 10; Dauno90, *n* = 11) died during observation time, with a median follow‐up of 23 months. The majority of deaths were infection‐related, while two were of cardiovascular origin. No significant associations were found between daunorubicin dose or post‐induction cardiac parameters (LVEF, hsTnT, NT‐proBNP) and overall survival by Spearman correlation, or relapse‐free survival by univariable Cox regression.

Of the 317 enrolled patients, LVEF values were available in 312 (98.4%) at baseline and 128 (40.4%) post‐induction. NT‐proBNP was assessed in 191 (60.3%) and 194 (61.2%) patients, and hsTnT in 142 (44.8%) and 140 (44.2%) at the respective time points. Missing data were primarily due to non‐performed assessments or the use of alternative biomarker methods (e.g., BNP instead of NT‐proBNP or non‐high‐sensitivity troponin assays).

### 3.1 Left Ventricular Systolic Function

**FIGURE 1:**
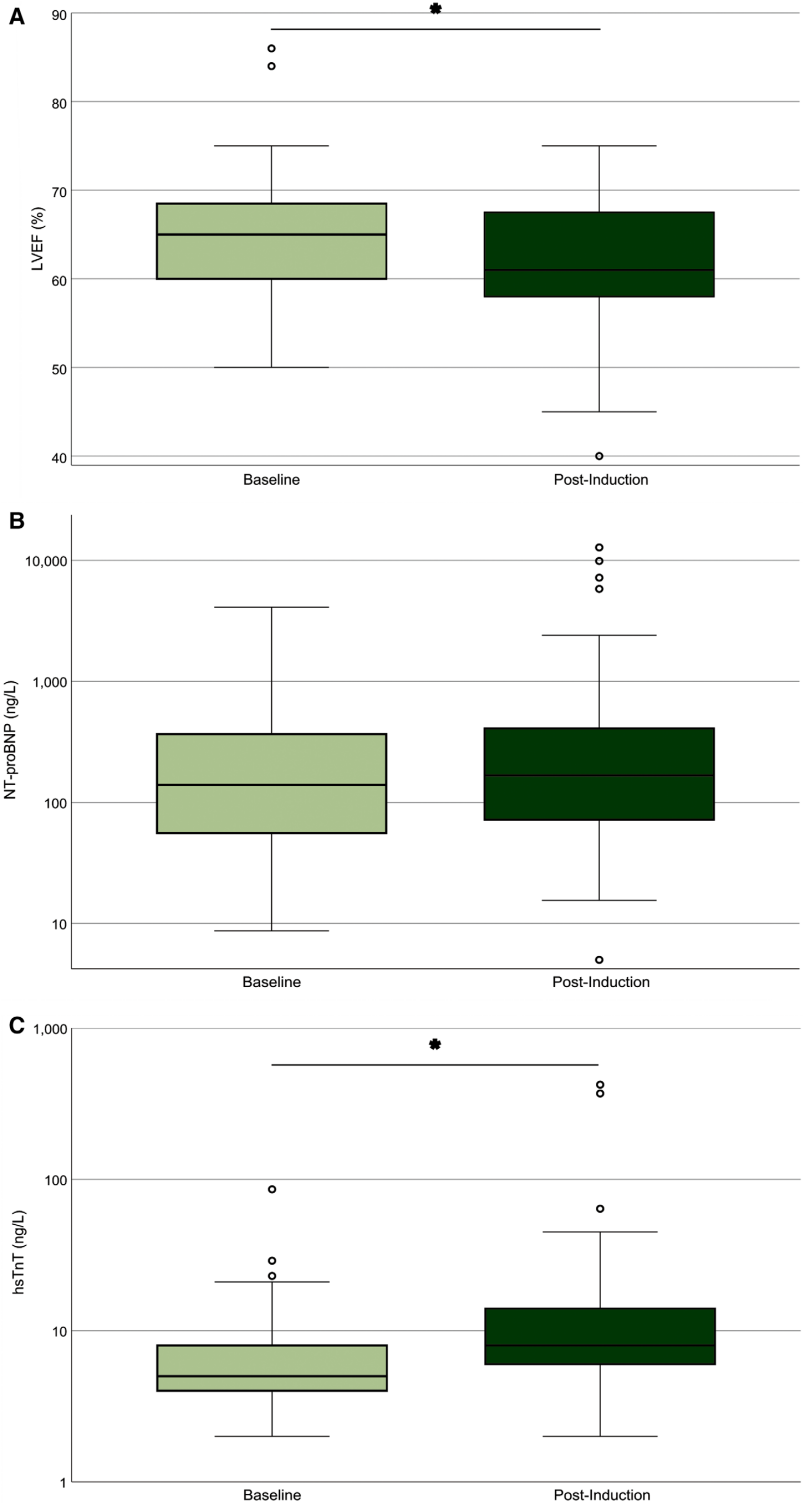
Cardiac function and biomarkers changed significantly from baseline to post‐induction therapy in AML patients. (A) Left ventricular ejection fraction (LVEF). (B) NT‐proBNP levels (ng/L, log scale). (C) High‐sensitivity troponin T (hsTnT, ng/L, log scale). Boxplots show the median (line), interquartile range (box), and whiskers extending to 1.5 × IQR. Outliers are displayed as individual points. LVEF declined significantly from baseline to post‐induction (*p* < 0.01). Median hsTnT levels increased from 5 to 8 ng/L (*p* < 0.01). The *Y*‐axis is logarithmic in (B and C) to account for skewed distributions. Asterisks (*) indicate statistically significant differences. [Color figure can be viewed at wileyonlinelibrary.com]

**FIGURE 2:**
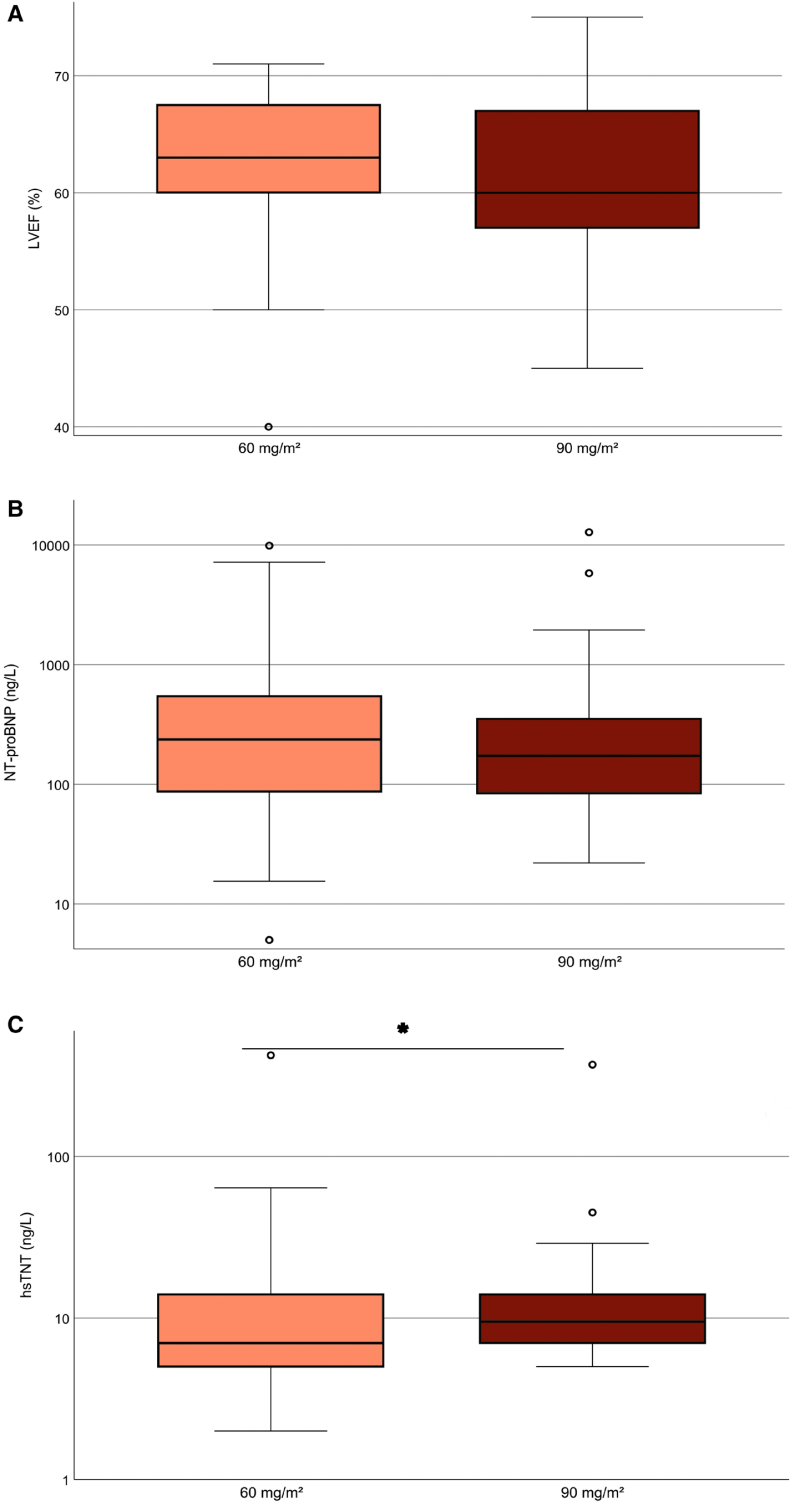
Post‐induction cardiac biomarkers differed by daunorubicin dose, with higher hsTnT levels in the 90 mg/m^2^ group. (A) Left ventricular ejection fraction (LVEF). (B) NT‐proBNP levels (ng/L, log scale). (C) High‐sensitivity troponin T (hsTnT, ng/L, log scale). Boxplots display the median (line), interquartile range (box), and whiskers extending to 1.5 × IQR. Outliers are shown as individual points. Median hsTnT levels were significantly lower in the Dauno60 group than in Dauno90 (*p* = 0.02). The *Y*‐axis is logarithmic in (B and C) to account for skewed distributions. Asterisks (*) indicate statistically significant differences. [Color figure can be viewed at wileyonlinelibrary.com]

At Day 15, the median LVEF had significantly declined from 65% [IQR 60–68.5] to 61% [IQR 58–67.75] in the total cohort (*n* = 128; *p* < 0.01; Figure 1A). Both treatment groups had an identical median LVEF of 65% at baseline. Post‐induction, median LVEF values were 63% [IQR 60–67.5] in Dauno60 and 60% [IQR 57–67] in Dauno90 (Figure 2A). This corresponds to a descriptive decline of 2 percentage points in the Dauno60 group and 5 percentage points in the Dauno90 group, but these differences were not statistically significant. Post‐induction LVEF did not differ between ELN risk groups (favorable 63% [IQR 60–68], intermediate 61% [IQR 57–68], adverse 60% [IQR 56–64]; *p* = 0.503). Overall, LVEF increased in 28 of 112 patients (25.0%), remained unchanged in 42 patients (37.5%), and decreased in 58 patients (51.7%) during induction therapy. A total of 20 patients (17.9%) experienced a LVEF decline ≥ 10 percentage points during induction therapy, comprising 9 of 52 patients (17.3%) in the Dauno60 group and 11 of 60 patients (18.3%) in the Dauno90 group. The lowest post‐induction LVEF observed was 40% (see outlier in Figure 2A). This patient had normal baseline cardiac parameters (LVEF 55%, NT‐proBNP 66 ng/L, hsTnT 4 ng/L) and developed febrile neutropenia during induction without documented cardiac complications.

### 3.2 NT‐proBNP

NT‐proBNP levels measured after induction therapy correlated negatively with LVEF at the same time point (*ρ* = −0.24, 95% CI –0.42 to −0.04; *p* = 0.02, *n* = 98), indicating that higher NT‐proBNP levels were associated with lower LVEF. NT‐proBNP levels increased during induction therapy from a baseline median of 139 [IQR 55.7–336] to 166.5 ng/L [IQR 72–408.1] at Day 15 (*n* = 150; Figure 1B), although this change did not reach statistical significance. Similarly, no significant difference was observed between treatment arms after therapy (Dauno60: 236.9 ng/L [IQR 86.7–554.5] vs. Dauno90: 173.0 ng/L [IQR 84–352.3]; *n* = 193; Figure 2B). Across ELN risk groups, median NT‐proBNP values were not significantly different (favorable 243.8 pg/mL [IQR 82.0–565.0], intermediate 165.0 pg/mL [84.9–343.0], adverse 219.0 pg/mL [63.6–554.0]).

### 3.3 hsTnT

hsTnT levels increased significantly from a median value of 5 ng/L [IQR 4–8] at baseline to a median of 8 ng/L [IQR 6–14] post‐induction across all patients (Wilcoxon test, *p* < 0.01; *n* = 117; Figure 1C). Median post‐induction hsTnT was significantly lower in the Dauno60 arm (7 ng/L [IQR 5–14]) than in the Dauno90 arm (9.5 ng/L [IQR 7–14]; Mann–Whitney test, *p* = 0.02; *n* = 140; Figure 2C). When stratified by ELN 2017 risk groups, no significant differences were detected (favorable 9.5 ng/L [6.0–14.0], intermediate 8.0 ng/L [5.5–14.0], adverse 11.5 ng/L [5.25–14.75]).

Three patients exhibited post‐induction hsTnT outlier values (Figure 2C). The highest value in the Dauno60 arm was 86 ng/L, observed in a patient with normal baseline cardiac parameters (hsTnT 4 ng/L, NT‐proBNP 66 ng/L) who developed febrile pharyngitis. In the Dauno90 arm, one patient with normal baseline values reached a peak hsTnT of 45 ng/L without documented cardiac complications. Another patient with arterial hypertension and elevated baseline biomarkers (hsTnT 86 ng/L, NT‐proBNP 638 ng/L) developed a maximum hsTnT of 370 ng/L in association with fever, edema, and intermittent dyspnea.

In exploratory analyses, post‐induction hsTnT correlated significantly with baseline hsTnT (*ρ* = 0.50; 95% CI, 0.35–0.63; *p* < 0.01), baseline NT‐proBNP (*ρ* = 0.21; 95% CI, 0.03–0.39; *p* = 0.03), daunorubicin dose (*ρ* = 0.20; 95% CI, 0.04–0.36; *p* = 0.02), age (*ρ* = 0.19; 95% CI, 0.02–0.36; *p* = 0.03), obesity (*ρ* = 0.19; 95% CI, 0.02–0.35; *p* = 0.03), and arterial hypertension (*ρ* = 0.22; 95% CI, 0.05–0.39; *p* = 0.01). Consistent with these findings, subgroup analyses revealed significantly elevated post‐induction hsTnT levels in patients with obesity and arterial hypertension. The effect of obesity was comparable to that of increased daunorubicin dosing, whereas hypertension was associated with a numerically greater hsTnT rise (Table 3). With respect to higher age, a similar trend was noted, although it did not reach statistical significance. These findings suggest that underlying conditions may contribute to early troponin release. The influence of cardiovascular risk factors on post‐induction hsTnT levels appeared similar across both treatment arms.

**TABLE 3:** Post‐induction hsTnT levels stratified by cardiovascular risk factors and daunorubicin dose.

	BMI ≥ 30 (*n* = 27)	BMI < 30 (*n* = 112)	AT (*n* = 32)	No AT (*n* = 108)	Dauno90 (*n* = 57)	Dauno60 (*n* = 60)	Age ≥ 55 (*n* = 46)	Age < 55 (*n* = 94)
hsTnT	11 [8–16]	8 [6–14]	13 [8–16]	8 [6–13]	9.5 [7–14]	7 [5–14]	10.5 [7–14]	8 [6–14]

*Note*: Values are medians [interquartile range]. Age is given in years, BMI in kg/m^2^, and hsTnT in ng/L. Between‐group differences were statistically significant (*p* < 0.05, Mann–Whitney test), except for age (*p* = 0.11).

Abbreviations: AT, arterial hypertension; BMI, body mass index; hsTnT, high‐sensitivity troponin T; *n*, number of patients.

## 4 Discussion

Cardiac monitoring of a large cohort of patients with a uniform diagnosis and predefined treatment allowed for a detailed evaluation of anthracycline‐induced cardiotoxicity during AML induction therapy. The prospective design of the DaunoDouble trial enabled early, standardized assessment of cardiac function and biomarker response to daunorubicin at two dose levels.

### 4.1 Early Systolic Changes and Dose Dependence of LVEF Decline

Anthracycline exposure during AML induction was associated with a measurable, dose‐dependent decline in left ventricular systolic function. Although absolute changes in LVEF were modest, a median reduction of 2 and 5 percentage points was observed at Day 15, corresponding to cumulative anthracycline exposures of 135 and 202.5 mg/m^2^ doxorubicin equivalents, respectively. These findings suggest that functional impairment may begin early during treatment. Notably, a 1.5‐fold increase in cumulative anthracycline dose was associated with an approximately 2.5‐fold greater decline in LVEF, supporting the concept of a nonlinear, dose‐dependent relationship between anthracycline exposure and cardiac dysfunction, as highlighted in the recent ESC guidelines on cancer therapy‐related cardiovascular toxicity [3].

These findings are in line with observations by Specchia et al., who reported a 4‐percentage‐point LVEF decline within seven days after induction at 225 mg/m^2^ cumulative anthracycline exposure in patients with hematologic malignancies [4]. Other, more heterogeneous oncology cohorts have demonstrated similar early LVEF reductions across cumulative anthracycline exposures between 200 and 240 mg/m^2^ doxorubicin equivalents (Table S1). Importantly, Cardinale et al. demonstrated that each 1% decrease in end‐of‐treatment LVEF was independently associated with a hazard ratio of 1.37 (95% CI, 1.33–1.42) for subsequent cardiotoxicity, defined as an LVEF decline > 10 percentage points and to a value < 50%, over a median follow‐up of 5.2 years [5]. Although long‐term follow‐up was not available in our study, the early LVEF declines observed may indicate an increased risk for subsequent cardiac dysfunction, underscoring the need for close cardiac surveillance after anthracycline‐based AML induction therapy.

### 4.2 NT‐proBNP Reflects Cardiac Strain but Lacks Dose Specificity

NT‐proBNP concentrations were already mildly elevated at baseline and showed a moderate increase after induction therapy, but did not differ significantly between daunorubicin dose arms. This variability is likely attributable to multiple influencing factors such as volume shifts, renal clearance, and systemic inflammation, as outlined in current heart failure guidelines [6]. Elevated baseline values, observed in a substantial proportion of patients, suggest that subclinical cardiac strain may already be present at diagnosis, potentially related to disease burden. The extent of NT‐proBNP increase observed in our cohort was modest and comparable to previous reports [7, 8], where similarly moderate post‐treatment elevations were noted. Although NT‐proBNP may reflect global myocardial stress, its role as a discriminative marker for dose‐dependent cardiac injury appears limited in this context.

### 4.3 High‐Sensitivity Troponin T as a Sensitive Marker of Early Cardiotoxicity

Among all assessed biomarkers, hsTnT most clearly differentiated the cardiac response to anthracycline exposure. Its early elevation in the higher‐dose group supports its role in detecting subclinical myocardial injury before overt functional decline. In our AML cohort, median hsTnT concentrations increased from 6.0 ng/L at baseline to 8.0 and 10.0 ng/L after cumulative doxorubicin‐equivalent doses of 135 and 202.5 mg/m^2^, respectively. Baseline hsTnT concentrations were elevated prior to treatment initiation and are consistent with values reported by Geiger et al., who found a median of 7.7 ng/L before anthracycline therapy [7], suggesting the presence of subclinical cardiac stress related to the underlying disease. In heterogeneous cancer populations, median hsTnT elevations between 8 and 19 ng/L have been observed following cumulative anthracycline‐equivalent doses of 150 to 240 mg/m^2^ (Table S2). However, the timing of troponin measurements in these studies differed from our trial, limiting direct comparability.

Although the median concentrations remained below established clinical decision thresholds, their consistent increase from baseline suggests a systematic effect indicative of early subclinical cardiotoxicity. Notably, one‐quarter of patients had post‐induction hsTnT levels above 14 ng/L, further supporting the potential relevance of these early biomarker changes in a vulnerable subgroup. While our study focused on early post‐treatment changes, the observed hsTnT elevations may already serve as an early indicator of long‐term cardiac dysfunction. Several prospective investigations in patients with hematologic malignancies have demonstrated that troponin elevations within days to weeks after anthracycline therapy predict subsequent LVEF decline [5, 7, 9]. In a landmark trial involving over 700 cancer patients, Cardinale et al. showed that pathological troponin levels within 72 h after chemotherapy completion were associated with a > 15 percentage point reduction in LVEF within 12 months in 70% of patients, compared to only 2.4% in those with normal values [5]. These findings highlight the prognostic relevance of early troponin release. Nonetheless, interpreting hsTnT levels remains challenging. Cardiac biomarker elevations may not solely reflect chemotherapy‐induced toxicity but can also be influenced by the underlying hematologic malignancy. Leukemia‐associated cardiac injury has been attributed to multiple mechanisms, including neoplastic infiltration or hemorrhage into myocardial tissue, hemodynamic changes such as hyperviscosity and anemia‐induced hypoxia, as well as cardiac or systemic inflammatory responses [10, 11]. Additionally, comorbidities such as chronic heart failure—even in clinically stable patients—may contribute to modest hsTnT elevations [12]. These considerations underscore the importance of evaluating hsTnT results in the broader clinical context. In addition, there is no clear consensus on the optimal timing for measurement [13], and no validated threshold currently exists to reliably guide individual risk assessment [2]. Despite these limitations, the available evidence suggests that even moderate hsTnT increases during anthracycline‐based AML induction therapy reflect meaningful myocardial toxicity and warrant structured follow‐up.

Our exploratory analyses also suggested modest associations between post‐induction hsTnT levels and clinical risk factors such as age, obesity, and hypertension. However, the impact of daunorubicin dose appeared consistent across these subgroups. In two of three patients with pronounced troponin elevations, concurrent symptoms indicative of systemic inflammatory or infectious complications were documented during induction therapy. These observations suggest that systemic inflammatory stress may contribute to or exacerbate myocardial injury in vulnerable individuals, underscoring the need to interpret hsTnT changes within the full clinical context and supporting the concept of a multifactorial pathogenesis of early cardiotoxicity in AML treatment.

### 4.4 Clinical Relevance in the Context of the DaunoDouble Trial

The observed cardiac effects must be interpreted alongside the primary outcomes of the DaunoDouble trial, which reported no significant differences in remission rates or survival between the two daunorubicin doses [1]. This provides critical context: intensifying anthracycline exposure increased cardiac biomarker burden without improving efficacy, supporting the use of 60 mg/m^2^ in standard induction. These findings also suggest that early cardiac monitoring may help identify patients at risk for cumulative cardiotoxicity or more pronounced early myocardial injury.

### 4.5 Limitations and Future Research Needs

This study assessed cardiac changes at a single post‐treatment time point, providing a snapshot rather than a trajectory of anthracycline‐related cardiotoxicity. However, prior studies have shown that early cardiac alterations after anthracycline exposure can predict subsequent dysfunction. Approximately 40% of the full cohort contributed to each cardiac parameter analysis due to missing or noncomparable data (e.g., BNP vs. NT‐proBNP; conventional vs. high‐sensitivity troponin assays), which limits generalizability. Furthermore, LVEF was assessed by echocardiography performed at the participating treatment centers without central review, entailing a risk of interobserver variability. LVEF quantification by centrally assessed cardiac magnetic resonance imaging is regarded as the gold standard and may, in future studies, allow for a more precise delineation of anthracycline‐related cardiac effects. Finally, information on clonal hematopoiesis of indeterminate potential (CHIP) was not available, precluding assessment of its potential impact.

Recent efforts to reduce anthracycline‐induced cardiotoxicity have focused on novel delivery systems such as liposomal daunorubicin/cytarabine [14] and lipophilic anthracycline derivatives currently under investigation [15]. In this context, dose adaptation of anthracyclines, as explored in this study, may serve as one component within a broader strategy to reduce cardiac burden without compromising efficacy. As part of such an approach, incorporating sensitive biomarkers such as hsTnT into routine cardiac monitoring protocols could help identify patients at increased risk and guide more personalized treatment decisions. Future research should further investigate the trajectory of cardiac marker changes and their predictive value for clinical outcomes such as heart failure, long‐term left ventricular dysfunction, or treatment interruptions. A key goal will be to define biomarker‐based thresholds that could trigger early cardioprotective interventions.

This study provides the first prospective evaluation of cardiac biomarker responses to 60 vs. 90 mg/m^2^ daunorubicin during AML induction. Anthracycline exposure was linked to an early decline in LVEF and a dose‐dependent rise in hsTnT. Our findings support early cardiac biomarker monitoring and dose adaptation within cardioprotective strategies, while long‐term follow‐up will be essential to confirm prognostic relevance and guide risk‐adapted treatment.

## Author Contributions

Conception and design: C.R., R.H., K.S.‐E., Jo.M., S.W.K., L.F., M.S., M.Kr., A.Ha., J.S., U.P., W.E.B., G.Eh., H.S., and M.B. Financial support: C.R. and Jo.M. Administrative support: C.R., J.‐H.M., Jo.M., L.F., F.F., U.P., W.E.B., J.M., H.S., and M.B. Provision of study materials or patients: C.R., B.St., J.‐H.M., M.Hä., M.Ha., M.Ka., K.S.‐E., A.N., A.B., C.D.B., Jo.M., E.J., D.N., A.Ho., G.He., T.Sz., Ch.Sm., L.F., S.K., M.S., J.S., J.M.M., F.S., C.T., W.E.B., J.M., H.S., M.B. Collection and assembly of data: C.R., B.St., Christoph Schliemann, J.‐H.M., N.A., R.H., M.Hä., M.Ha., M.Ka., B.W., K.S.‐E., A.N., C.D.B., Jo.M., E.J., D.N., S.W.K., S.S., A.Ho., G.He., T.Sz., A.R., Christoph Schmid, L.F., S.K., M.S., M.Kr., F.F., A.Ha., J.M.M., F.S., U.P., C.T., W.E.B., and J.M. Data analysis and interpretation: Ch.R., S.M.D., M.Kr., K.S.‐B. Manuscript drafting and writing: S.M.D. and C.R. were responsible for drafting the initial manuscript. All authors contributed to critical revision, approved the final version, and are accountable for all aspects of the work.

## Funding

This work was supported by institutional funding from Technische Universität Dresden. No external grants or awards were received.

## Ethics Statement

The trial was conducted under the sponsorship of Technische Universität Dresden, Germany, with financial support provided by its Medical Faculty. The study was performed in accordance with the Declaration of Helsinki and approved by the Ethics Committee of the Faculty of Medicine, Technische Universität Dresden, Germany.

## Consent

Written informed consent was obtained from all patients prior to study enrollment.

## Conflicts of Interest

C.R. has received honoraria from AbbVie, Astellas, Bristol‐Meyer‐Squibb, Daiichi Sankyo, Jazz, Janssen, Novartis, Otsuka, Pfizer, Servier, and institutional research funding from AbbVie, Astellas, Novartis, Pfizer. Christoph Schliemann has received honoraria and/or served on advisory boards for AbbVie, Astellas, AstraZeneca, Bristol‐Myers Squibb, Daiichi Sankyo, Delbert Laboratories, Jazz Pharmaceuticals, Novartis, Otsuka, Pfizer, and Roche; institutional research support from Jazz Pharmaceuticals; and congress/travel grants from AbbVie, Bristol‐Myers Squibb, Jazz Pharmaceuticals, and Pfizer. The other authors declare no conflicts of interest.

## Supporting information


**Table S1:** Literature overview: LVEF reduction following anthracycline therapy.

**Table S2:** Literature overview of troponin concentration following anthracycline therapy.

## Data Availability

Deidentified individual participant data underlying the reported results will be made available upon reasonable request from the corresponding author. Data will become accessible 3 months after publication and remain available for 3 years. Requests must include a methodologically sound proposal and will require approval by the corresponding author. For inquiries regarding the availability of original data, please contact the corresponding author. No additional documents (e.g., study protocol or statistical analysis code) will be shared.
