# Supplementary material for: Randomized Comparison of Cardiotoxicity With 60 Versus 90 mg Daunorubicin in AML Induction Therapy
Source: Am J Hematol. 2026 Jan 5;101(3):512–20. doi: 10.1002/ajh.70160 (PMC12869000; doi:10.1002/ajh.70160)
Supplement: Supplementary file 1 — Table S1: Literature overview: LVEF reduction following anthracycline therapy. [file AJH-101-512-s002.docx]

Supplementary Table S1 Literature overview: LVEF reduction following anthracycline therapy.

| Reference | Anthracycline Dose | LVEF Reduction | Primary Disease | Timepoints of Measurement |
| --- | --- | --- | --- | --- |
| (9) | 118 178 | 0.7 2.0 | Heterogeneous | 6 weeks 12 weeks |
| Present study | 135 202.5 | 2.0 5.0 | Hematologic | 10 days 10 days |
| (7) | 200 | 1.5 | Heterogeneous | 3 months |
| (4) | 225 | 5.0 | Hematologic | 7 days |
| (16) | 240 | 4.0 | Heterogeneous | 3 months |
| (5) | 299 359 | 2.0 5.0 | Heterogeneous | 1 to 48 months |

Legend: Anthracycline dose refers to the mean cumulative dose, expressed in mg/m² as doxorubicin equivalents. LVEF reduction is presented as absolute change in percentage points. Primary disease indicates the underlying malignancy; timepoint refers to the interval after completion of anthracycline therapy. Reference (16), B. C. Drafts, K. M. Twomley, R. D'Agostino, et al., “Low‐To‐Moderate‐Dose Anthracycline‐Based Chemotherapy Is Associated With Early Noninvasive Imaging Evidence of Subclinical Cardiovascular Disease,” *JACC Cardiovascular Imaging* 6, no. 8 (2013): 877–885.
