# Supplementary material for: Randomized Comparison of Cardiotoxicity With 60 Versus 90 mg Daunorubicin in AML Induction Therapy
Source: Am J Hematol. 2026 Jan 5;101(3):512–20. doi: 10.1002/ajh.70160 (PMC12869000; doi:10.1002/ajh.70160)
Supplement: Supplementary file 2 — Table S2: Literature overview of troponin concentration following anthracycline therapy. [file AJH-101-512-s001.docx]

Supplementary Table S2. Literature overview of troponin concentration following anthracycline therapy.

| Reference | Anthracycline Dose | cTn Concentration | Primary Disease | Timepoint of Measurement |
| --- | --- | --- | --- | --- |
| (17) | 0 180 | 1.3 11.7 | Breast cancer | Baseline undefined |
| (18) | 0 240 | 1.3 11.7 | Breast cancer | Baseline 3 months |
| (19) | 0 240 | 1.4 19.1 | Heterogeneous | Baseline 4 weeks |
| Present study | 0 135 202.5 | 6.0 8.0 10.0 | Hematologic | Baseline 10 days 10 days |
| (7) | 0 150 | 7.7 8.9 | Heterogeneous | Baseline 7 days |

Legend: Anthracycline dose refers to the mean cumulative dose, expressed in mg/m² as doxorubicin equivalents. cTn concentration is reported as median in ng/L. Primary disease indicates the underlying malignancy; timepoint refers to the interval after completion of anthracycline therapy. Abbreviations: cTn, cardiac troponin. Reference (17), B. Ky, M. Putt, H. Sawaya, et al., “Early Increases in Multiple Biomarkers Predict Subsequent Cardiotoxicity in Patients With Breast Cancer Treated With Doxorubicin, Taxanes, and Trastuzumab,” *Journal of the American College of Cardiology* 63, no. 8 (2014): 809–816. Reference (18), H. Sawaya, I. A. Sebag, J. C. Plana, et al., “Assessment of Echocardiography and Biomarkers for the Extended Prediction of Cardiotoxicity in Patients Treated With Anthracyclines, Taxanes, and Trastuzumab,” *Circulation. Cardiovascular Imaging* 5, no. 5 (2012): 596–603. Reference (19), A. H. Blaes, A. Rehman, D. M. Vock, et al., “Utility of High‐Sensitivity Cardiac Troponin T in Patients Receiving Anthracycline Chemotherapy,” *Vascular Health and Risk Management* 11 (2015): 591–594.
